# Transmission of social status drives cooperation and offspring philopatry

**DOI:** 10.1098/rspb.2023.1314

**Published:** 2023-11-29

**Authors:** António M. M. Rodrigues, Andy Gardner

**Affiliations:** ^1^ Department of Integrative Biology, University of California, Berkeley, CA 94720, USA; ^2^ Department of Bioengineering, Stanford University, Stanford, CA 94305, USA; ^3^ Department of Ecology & Evolutionary Biology, Yale University, New Haven, CT 06511, USA; ^4^ School of Biology, University of St Andrews, St Andrews KY16 9TH, UK

**Keywords:** kin competition, kin selection, non-genetic inheritance, reproductive value, major transitions

## Abstract

The evolution of cooperation depends on two crucial overarching factors: relatedness, which describes the extent to which the recipient shares genes in common with the actor; and quality, which describes the recipient's basic capacity to transmit genes into the future. While most research has focused on relatedness, there is a growing interest in understanding how quality modulates the evolution of cooperation. However, the impact of inheritance of quality on the evolution of cooperation remains largely unexplored, especially in spatially structured populations. Here, we develop a mathematical model to understand how inheritance of quality, in the form of social status, influences the evolution of helping and harming within social groups in a viscous-population setting. We find that: (1) status-reversal transmission, whereby parental and offspring status are negatively correlated, strongly inhibits the evolution of cooperation, with low-status individuals investing less in cooperation and high-status individuals being more prone to harm; (2) transmission of high status promotes offspring philopatry, with more cooperation being directed towards the higher-dispersal social class; and (3) fertility inequality and inter-generational status inheritance reduce within-group conflict. Overall, our study highlights the importance of considering different mechanisms of phenotypic inheritance, including social support, and their potential interactions in shaping animal societies.

## 1. Introduction

In order for altruism to evolve, social interactions must occur between related individuals [1,2], as relatedness enables altruists to derive inclusive fitness through copies of their genes that are located in the bodies of social partners [1–4]. Population viscosity is one of the main mechanisms understood to generate relatedness among social partners [1,2,5–9]. In viscous populations, individuals tend to remain near their place of birth, and therefore neighbours are more likely to carry genes in common than random individuals in the population [1]. Unlike other mechanisms for generating relatedness, population viscosity does not require that organisms possess special adaptations that enable discrimination between kin and non-kin, and it therefore has the potential to drive the evolution of altruism across the whole diversity of life, from microbes to plants to vertebrates [2].

More recently, there has been a growing interest in understanding the influence of population viscosity on altruism in class-structured populations [4], such as when some individuals are born into favourable conditions and are destined to enjoy greater success regardless of their genetic abilities [4]. This literature has explored various factors, such as spatiotemporal variation in local resource availability [10] and group size [11], quality of group members [12], individual and group age [13,14], and intergroup conflict and cooperation [9,15]. This work has shown that an individual's class can influence their social behaviour by impacting their future reproductive and survival prospects, leading to correlations between the fitness costs and benefits of cooperation and an individual's condition [4], which, unlike relatedness, may even explain variation in cooperation within clonal groups [12,16,17]. For reasons of simplicity and analytical tractability, these studies of class-structured populations have assumed that parent and offspring classes are causally independent, neglecting mechanisms of class inheritance. However, this assumption will hardly ever be met in the real world. Epigenetic effects—including those that are socially mediated—in which the condition of an ancestor is a determinant of the quality of a descendant, independently of the alleles that they share in common, occur in practically every species in which they have been looked for, from yeast to waterfleas to primates [18–27].

Of particular relevance in a wide range of species is variation in an individual's condition associated with their status within a social hierarchy. Socially stratified societies exist in most animal taxa, being especially well-known among insects, mammals, primates and humans [28,29], and where socially stratified species occur they tend to be ecologically important—for example, the hugely successful societies of ants and humans that each account for comparable portions of the Earth's total biomass [30]. Status and status-reversal transmission has been documented in multiple taxa [27], including birds [31,32], mammals [33–37], monkeys [38–43], great apes [19,44] and recent human populations [45], where it has been found to have substantial impacts on individual fitness, group social dynamics and the potential to influence evolutionary trajectories are higher levels of biological organization [27]. Despite this, the ramifications of rank inheritance versus rank-reversal inheritance for the evolution of cooperation remain obscure.

Here, we develop a mathematical model to study the evolution of helping and harming behaviour in an explicit demographic context in which individuals vary in their fecundity according to social status that is transmitted with greater or lesser fidelity from parent to offspring. We investigate how individuals may be favoured to adjust their social behaviours conditionally according to their status. We consider scenarios in which dispersal is treated as a fixed parameter. Additionally, we investigate cases where dispersal is an evolving phenotype that may or may not be adjusted conditionally according to status. Our analysis highlights the role of parental and offspring condition—and the correlation between these—in modulating the evolution of cooperation.

## 2. Model and analysis

### (a) Life cycle

We assume an infinite island model [46] and a population of asexually reproducing haploid individuals in which group members differ with regards to their social status [12,47,48]. We assume that each patch contains *n*_j_ breeding individuals of j-status, where j ∈ Ω = {H,L}, with H denoting ‘high-status’ and L denoting ‘low-status’. The fecundity of j-status individuals is *f*_j_(**x**_h_,**Y**_h_), which depends on their own social behaviour **x**_h_ = (*x*_H,h_, *x*_L,h_), and on the social behaviour of their social partners **Y**_h_ = (*Y*_H,h_,*Y*_L,h_), where the subscript ‘h’ denotes a phenotype on the helping-harming continuum. We define the level of reproductive inequality as *I* = 1 − (*f*_L_(**x**_h_,**Y**_h_)/*f*_H_(**x**_h_,**Y**_h_)), with *I* = 0 when *f*_L_(**x**_h_,**Y**_h_) = *f*_H_(**x**_h_,**Y**_h_), and *I* ≈ 1 when *f*_L_(**x**_h_,**Y**_h_) ≪ *f*_H_(**x**_h_,**Y**_h_). Following reproduction, a fraction 1 − *z*_j,d_ of the j-born offspring remains in the local patch, while a fraction *z*_j,d_ disperses independently to random patches elsewhere in the population, where the subscript ‘d’ denotes the dispersal phenotype. Dispersing individuals suffer a mortality cost *k*, resulting in each individual's survival with a probability 1 − *k*. Offspring, whether native or immigrant, compete for the available breeding sites, with each offspring being equally likely to compete for the high-status and low-status positions. We assume that mothers influence the outcome of competitive interactions among offspring, such that native offspring, either high- or low-born, have a home-ground competitive advantage over dispersed offspring [9,27,44,49–51]. In the appendix, we explore the less common contrasting scenario in which dispersed offspring have a competitive advantage over non-dispersing offspring (reviewed in [27]). We assume that non-dispersing high-born offspring are more likely to win the high-status breeding position under rank inheritance, and non-dispersing low-born offspring are more likely to win the high-status breeding position under rank-reversal inheritance. Specifically, we assume that: the probability that high-born philopatric offspring win the high-status breeding position is weighted by a factor $m_{H\to H}^{\phi }=1-M$; the probability that low-born philopatric offspring win the high-status breeding position is weighted by a factor $m_{L\to H}^{\phi }=M$; and the probability that each of the other migrant juveniles competing for that position wins the position is weighted by a factor $m_{j\to l}^{\delta }=M_{o}$. As a result, there is unbiased inheritance of status—‘level playing field’—when *M* = 1/2, maternal inheritance of status—‘social hypo-mobility’—when *M* < 1/2, where parental and offspring status become positively correlated, and reversed inheritance of status—‘social hyper-mobility’—when *M* > 1/2, where parental and offspring status become negatively correlated, with *M*_o_ = 1 − *M* when *M* > 1/2 and *M*_o_ = *M* when *M* < 1/2. We assume no such bias in relation to competition for the low-status breeding position. Hence, $m_{i\to L}^{\phi }=m_{i\to L}^{\delta }=1$, with i ∈ Ω. Following the competition stage, all individuals occupying breeding positions become reproductively mature, and the rest perish, bringing the population to the beginning of the life cycle.

### (b) Selection gradient and inclusive fitness effect

To analyse the model, we employ the neighbour-modulated approach to kin selection, which enables the calculation of selection gradients in class-structured populations [4,52–54]. Here, we present a concise overview of the derivations for obtaining the selection gradients. Equations (2.4) and (2.5) summarize the main results for readers wishing to skip the derivations. A comprehensive analysis of the model is provided in the appendix (electronic supplementary material). The total fitness in the population is given by *w*(**x**,**Y**,**z**) = **vA**(**x**,**Y**,**z**)**u** where: **v** is the row-vector of individual reproductive values; **u** is the column-vector of stable class frequencies; **A**(**x**,**Y**,**z**) is the (full gametic) fitness matrix; and **x**, **Y** and **z** denote the phenotype of focal individuals, the mean phenotype within classes in a focal patch, and the population mean phenotype within classes, respectively, for both helping-harming and dispersal behaviour. The vectors **v** and **u** are the dominant left- and right-eigenvectors, respectively, of the normal fitness matrix A*, where A* assumes a neutral population (=**A**(**z**,**z**,**z**)). The fitness matrix is given by

2.1 $$\begin{array}{rl} A(x,Y,z)= & \underset{A^{\phi }(x,Y,z)}{\underbrace{\left(\begin{array}{cc} w_{H\to H}^{\phi }(x,Y,z) & w_{L\to H}^{\phi }(x,Y,z)\\ w_{H\to L}^{\phi }(x,Y,z) & w_{L\to L}^{\phi }(x,Y,z) \end{array}\right)}}\\ & +\underset{A^{\delta }(x,Y,z)}{\underbrace{\left(\begin{array}{cc} w_{H\to H}^{\delta }(x,Y,z) & w_{L\to H}^{\delta }(x,Y,z)\\ w_{H\to L}^{\delta }(x,Y,z) & w_{L\to L}^{\delta }(x,Y,z) \end{array}\right)}}, \end{array}$$

where $A^{\phi }(x,Y,z)$ is the ‘philopatric-offspring’ fitness matrix, which includes the components of fitness generated through philopatric offspring, and $A^{\delta }(x,Y,z)$ is the ‘dispersed-offspring’ fitness matrix, which includes the components of fitness generated through dispersed offspring. Each column of the fitness matrices represents the class of a focal recipient, and the entries within the column the genetic contribution of the focal recipient to the different classes, where

2.2 $$\begin{array}{c} w_{i\to j}^{\phi }(x,Y,z)=\frac{\;f_{i}(x_{h},Y_{h})(1-x_{i,d})m_{i\to j}^{\phi }}{\Sigma_{p\in \Omega }n_{p}f_{p}(Y_{h},Y_{h})(1-Y_{p,d})m_{p\to j}^{\phi }+\Sigma_{p\in \Omega }n_{p}f_{p}(z_{h},z_{h})z_{p,d}(1-k)m_{p\to j}^{\delta }}n_{j},\;\mathrm{and}\\ w_{i\to j}^{\delta }(x,Y,z)=\frac{\;f_{i}(x_{h},Y_{h})x_{i,d}m_{i\to j}^{\delta }(1-k)}{\Sigma_{p\in \Omega }n_{p}f_{p}(z_{h},z_{h})(1-z_{p,d})m_{p\to j}^{\phi }+\Sigma_{p\in \Omega }n_{p}f_{p}(z_{h},z_{h})z_{p,d}(1-k)m_{p\to j}^{\delta }}n_{j},\;\; \end{array}$$

represent the contribution of a focal class-i recipient to class-j through philopatric and dispersed offspring, respectively. The phenotype *x*_i__,__d_ denotes the dispersal rate of the focal class-i mother, while the phenotype *Y*_p__,d_ denotes the average dispersal rate of the local class-p mothers. The numerators represent the number of offspring produced by a focal class-i female, her capacity to transmit her status to her offspring, and the number of available positions for a particular status class. The denominators contain all the offspring that compete for the status class. The selection gradient is given by the derivative of total fitness with respect to the breeding value at the locus of interest, d*w*(**x**,**Y**,**z**)/d*ℊ*. We expand this derivative to obtain:

2.3 $$\begin{array}{rl} \frac{dw(x,Y,z)}{dg}= & v\left(\frac{\partial A^{\phi }(x,Y,z)}{\partial x_{\alpha }}+\frac{\partial A^{\phi }(x,Y,z)}{\partial Y_{\alpha }}\circ R\right)u\\ & +v\left(\frac{\partial A^{\delta }(x,Y,z)}{\partial x_{\alpha }}+\frac{\partial A^{\delta }(x,Y,z)}{\partial Y_{\alpha }}\circ R\right)u, \end{array}$$

where **R** = (**R**_α__→H_ **R**_α__→L_) is the relatedness matrix, with the dimension of **A**, in which the columns **R**_α__→H_ and **R**_α__→L_ give the relatedness between the class-α actors and a class-i recipient, *R*_α__→i_ with i ∈ Ω, and where ‘∘’ denotes the entrywise product binary operation. Without loss of generality, we set the baseline fecundity of high-status breeders to one, such that the baseline fertility of low-status breeders becomes 1 − *I*, with 0 ≤ *I* ≤ 1. We assume that helping (or harming) carries a fecundity cost for α-status actors and confers a fecundity benefit (or cost) on the ρ-status social partners, with α ≠ ρ. To quantify the marginal cost and the neighbour-modulated benefit of the behaviour, we use the partial derivatives *c*_α_ = −∂*f*_α_(**x**_h_,**Y**_h_)/∂*x*_α,h_ and *b*_ρ__←α_ = ∂*f*_ρ_(**x**_h_,**Y**_h_)/∂*Y*_α__,h_, respectively [12]. It is important to highlight that the ‘neighbour-modulated benefit’ *b*_ρ__←α_ specifically captures the influence of class-α actors on a focal class-ρ recipient. In order to determine the inclusive fitness effect of the behaviour, we must express the selection gradient in terms of the ‘inclusive-fitness benefit’, denoted as *b*_α__→ρ_, which quantifies the effect of a single class-α actor on the fertility of class-ρ recipients [12]. This rearrangement of the neighbour-modulated selection gradient enables the derivation of Hamilton's rule, establishing the condition for the evolution of the behaviour based on the inclusive fitness effect:

2.4 $$\begin{array}{rl} & \;\;\;\;\;\;-c_{\alpha }V_{\alpha }+b_{\alpha \to \rho }V_{\rho }R_{\alpha \to \rho }\\ & -\sum_{l\in \Omega }\left(b_{\alpha \to \rho }O_{\rho \to l}^{\phi }-c_{\alpha }O_{\alpha \to l}^{\phi }\right)\sum_{\;j\in \Omega }n_{j}w_{j\to l}^{\phi }v_{l}R_{\alpha \to j}>0, \end{array}$$

where *V*_α_ (or *V*_ρ_) represents the reproductive value of the actor's (or recipient's) offspring, and $O_{\rho \to l}^{\phi }$ (or $O_{\alpha \to l}^{\phi }$) represents the probability of choosing a ρ-born (or α-born) offspring competing for l-status positions in its native patch, and where we exclude the argument from the notation of functions to simplify the presentation. The left-hand side of inequality (2.4) describes the inclusive fitness of a class-α actor. The actor produces *c*_α_ fewer offspring, each representing a decrement *V*_α_ in her fitness. However, the recipients produce *b*_α__→ρ_ extra offspring, each representing an increment *V*_ρ_ in their personal fitness. Finally, helping creates $b_{\alpha \to \rho }O_{\rho \to l}^{\phi }-c_{\alpha }O_{\alpha \to l}^{\phi }$ additional offspring securing local l-status positions (with l ∈ Ω), which displaces class-j mothers' (*n*_j_) philopatric offspring $\left(w_{j\to l}^{\phi }\right)$, whose reproductive values would be *v*_l_ if they were not displaced by the additional offspring. All fitness effects must be depreciated by the coefficient of relatedness between the actor and recipients (*R*_α__→j_). The condition for the evolution of dispersal is given by

2.5 $$-V_{j}^{\phi }+V_{j}^{\delta }+\underset{l\in \Omega }{\sum }P_{j\to l}^{\phi }\underset{p\in \Omega }{\sum }(n_{p}w_{p\to l}^{\phi }v_{l}R_{j\to p})>0,$$

where $V_{j}^{\phi }$ (or $V_{j}^{\delta }$) is the reproductive value of a non-dispersing (or dispersing) j-born offspring, and $P_{j\to l}^{\phi }$ is the probability of choosing a non-dispersing j-born offspring among all offspring competing for l-status positions. The left-hand side of inequality (2.5) describes the inclusive fitness effect of dispersal for a focal class-j female. Dispersal involves a fraction of previously non-dispersing offspring leaving the patch, with these offspring now generating the reproductive value of dispersing offspring, $V_{j}^{\delta }$, rather than that of non-dispersing offspring, $V_{j}^{\phi }$. With probability $P_{j\to l}^{\phi }$, the additional dispersing offspring create new local breeding opportunities, which, with probability $w_{p\to l}^{\phi }$, are used by offspring of local females (*n*_p_), who generate a reproductive value *v*_l_. This effect must be depreciated by the relatedness between the focal female and her social partners in class-p, *R*_j__→p_.

### (c) Potential for helping and optimal dispersal rates

We express the selection pressures acting on the helping (or harming) behaviour in terms of the potential for helping (denoted by *h*; [12]), this being the ratio of marginal fecundity effects at which the individual breaks even. Specifically, the potential for helping of α-status individuals is defined as *h*_α_ = *c*_α_/*b*_α__→ρ_, in which a higher *h* means more helping is favoured and a lower *h* means less helping is favoured – and negative *h* means that harming is favoured. To determine the optimal levels of dispersal, *z*_d_*, we employ an iterative algorithm that involves substituting small-effect alternative strategies for the resident strategy (i.e. *x*_d_ = *z*_d_ + *δ*, where *δ* → 0). This process is repeated until the fitness gradient(s) of dispersal reach equilibrium and selection for higher or lower levels of dispersal vanishes (e.g. [13,55,56]).

## 3. Inheritance of status and the evolution of behaviour

We aim to explore how access to status affects cooperation, focusing on an offspring's ability to acquire high-ranking positions within their group. We investigate both maternal rank inheritance (*M* < 1/2), where high-born offspring have privileged access to status, and rank-reversal inheritance (*M* > 1/2), where low-born offspring have privileged access to status.

### (a) Inheritance of status promotes cooperation

**Figure 1.**
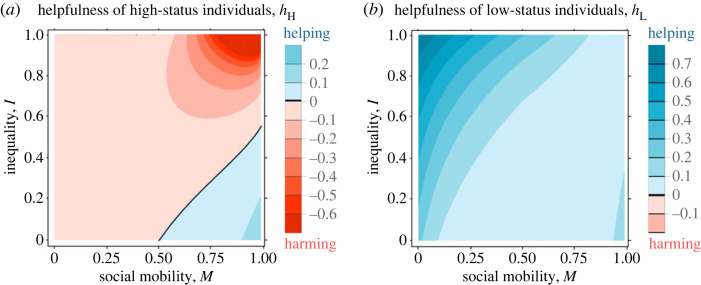
Maternal rank inheritance tends to promote helping by low-status individuals. Potential for helping by high- and low-status individuals (i.e. *h*_H_ and *h*_L_) as a function of the degree of inequality (*I*) and the degree of inter-generational rank inheritance (*M*). High inequality coupled with reversed-rank inheritance promotes extreme levels of harming by high-status individuals and lower levels of helping by low-status individuals. Parameter values: *k* = 0.5, *n*_H_ = 1, *n*_L_ = 2, *z*_j,d_ = 0.5.

**Figure 2.**
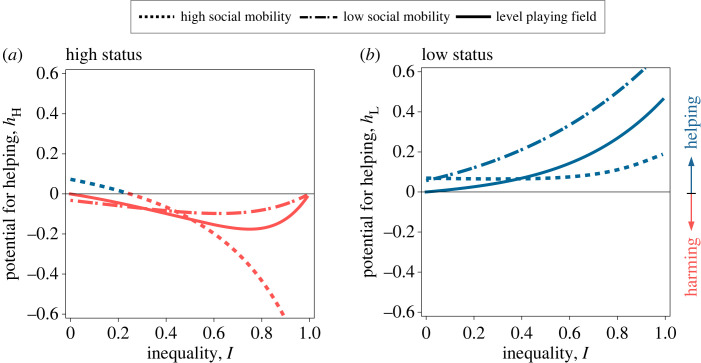
Maternal rank inheritance gives rise to more cooperative societies, with high-status individuals investing less in harming and low-status individuals investing more in helping. Potential for helping by high- and low-status individuals (i.e. *h*_H_ and *h*_L_; (*a*) and (*b*), respectively) as a function of inequality (*I*) for a level playing field (i.e. *M* = 0; solid lines), maternal rank inheritance (i.e. *M* = 0.25; dashed lines) and reversed-rank inheritance (i.e. *M* = 0.75; dotted lines). In a level playing field, high-status (or low-status) mothers invest more into harming (or helping). Reversed-rank inheritance can either promote cooperation (when inequality in fertility is lower), or selfishness (when inequality in fertility is higher). Parameter values: *k* = 0.5, *n*_H_ = 1, *n*_L_ = 2, *z*_j,d_ = 0.5.

We start by treating offspring dispersal as a model parameter that is independent of parental status (i.e. for all j ∈ Ω, *z*_j,d_ = *z*_U,d_), before considering the consequences of allowing dispersal to evolve. When there is unbiased inheritance of status (i.e. *M* = 1/2), we find that while reproductively egalitarian populations (i.e. *I* = 0) favour neither helping nor harming by any individual, populations that are characterized by some disparity in reproductive quality (i.e. *I* > 0) favour harming by high-status individuals and helping by low-status individuals (figures 1 and 2). Helping directly increases the inclusive fitness of the actor through the production of related offspring. However, helping also has a negative impact on the inclusive fitness of the actor, because a fraction of the additional offspring remains in the local patch and displace some of the actor's offspring and other related offspring. In reproductively egalitarian societies, the extra amount of competition imposed by helping exactly cancels its benefits, and therefore neither helping nor harming is favoured [12,57]. Disparities in reproductive inequality (i.e. *I* > 0), however, decouple these two effects, and hence disrupt their mutual cancellation (cf. [58,59]). This is because high-status individuals contribute more to the pool of local offspring. As a result, the additional competition created by helping falls disproportionally among high-born offspring, which reduces the incentive for high-status mothers to invest into helping. By contrast, low-status mothers contribute less to the pool of local offspring and therefore suffer less from the negative effects of additional offspring, which motivates them to invest more into helping. The combination of these two effects—harming by high-status individuals and helping by low-status individuals—means that the high fecundity of high-status individuals will become even higher and the low fecundity of low-status individuals will become even lower, and therefore the initial inequality tends to increase.

#### Main prediction 1

Mechanisms leading to rank inheritance (*M* < 1/2) increase the average level of cooperation within groups, with high-status individuals allocating fewer resources to the harmful suppression of the partners' reproductive output, and with low-status individuals investing more resources in a partner's reproductive output (figures 1 and 2).

Full or even partial transmission of hierarchical status promotes helping behaviour among low-status individuals, even in the absence of inequality that translates into differential fecundity (i.e. *I* = 0). This is because it is harder for low-status offspring to gain access to the local breeding sites, making it advantageous for them to invest into high-born offspring who can claim breeding sites more easily. Intuitively, one might expect high-status individuals to invest more in suppressing competition because their offspring are more valuable. However, this is not the case. First, the immediate cost of harming is born in terms of offspring, and therefore more valuable offspring also raises the cost of harming. Second, the benefit of harming is relatively low. An actor's primary benefit of harming social partners and destroying their offspring is to ease competition for the actor's own offspring. However, the actor's offspring are already superior competitors, so destroying weaker low-born offspring with limited competitive ability offers little fitness benefits to high-status individuals.

#### Main prediction 2

Rank-reversal inheritance typically leads to the evolution of harming by high-status individuals and helping by low-status individuals (figures 1 and 2).

In general, low-status individuals allocate fewer resources to helping but never come to invest in harming, as we could expect if there was complete reversal of social roles. This is because although low-born offspring are more likely to gain access to high-status positions, these positions offer fewer rewards because the offspring of high-status individuals are more likely to become low-status individuals due to rank-reversal inheritance. Overall, across a wide range of parameter values, we consistently observe a decline in the average level of cooperative behaviour within the social group. High-status individuals are now selected to allocate a larger share of their resources to harming behaviour (i.e. *h*_H_ ≪ 0), especially under extreme inequality (higher *I*), while low-status individuals are favoured to allocate a smaller share of their resources to helping behaviour (i.e. *h*_L_ → 0).

### (b) Transmission of status and mean offspring dispersal behaviour

**Figure 3.**
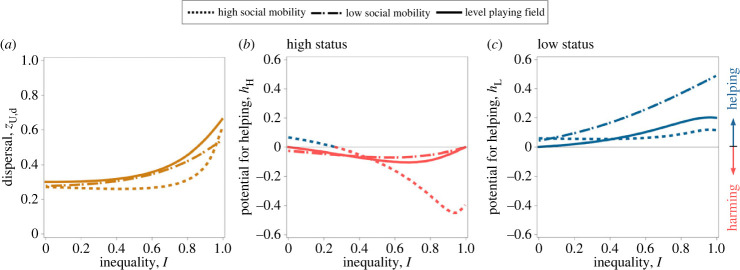
Natural selection favours intermediate levels of dispersal. Optimal dispersal rates (*z*_U,d_; (*a*)), potential for helping by high- and low-status individuals (i.e. *h*_H_ and *h*_L_, (*b*) and (*c*), respectively) as a function of inequality (*I*) for a level playing field (i.e. *M* = 0; solid lines), maternal rank inheritance (i.e. *M* = 0.25; dashed lines) and reversed-rank inheritance (i.e. *M* = 0.75; dotted lines). Parameter values: *k* = 0.5, *n*_H_ = 1, *n*_L_ = 2.

In our initial analysis, we have seen that limited dispersal leads to asymmetric kin competition between high- and low-status individuals, a factor that mediates their divergent social roles. However, the exact level of dispersal influences the role that kin competition plays in the expression of parental behaviour, with relatively high levels of dispersal mitigating the amount of competition among native related offspring (i.e. kin competition), and relatively lower levels of dispersal exacerbating this. Therefore, to determine the degree of social differentiation within a social group, it is important to understand the exact levels of dispersal favoured by natural selection when dispersal is allowed to evolve. If dispersal is itself able to evolve, and independent of parental status (i.e. for all j ∈ Ω, *z*_j,d_ = *z*_U,d_), we find that individuals are selected to produce a moderate number of dispersers (i.e. *z*_U,d_ ≈ 0.5), which generates a significant level of competition among native related offspring for breeding resources (figure 3). As a result, we find marked social differentiation within the social group, in which higher- and lower-status individuals invest substantial resources into harming and helping behaviours, respectively. We find that the degree of status transmission has little impact upon the optimal levels of dispersal. Both strong rank inheritance and strong rank-reversal inheritance favour slightly lower rates of offspring dispersal relative to those favoured under egalitarian transmission of privilege (figure 3*a*).

### (c) Maternal support promotes offspring philopatry

**Figure 4.**
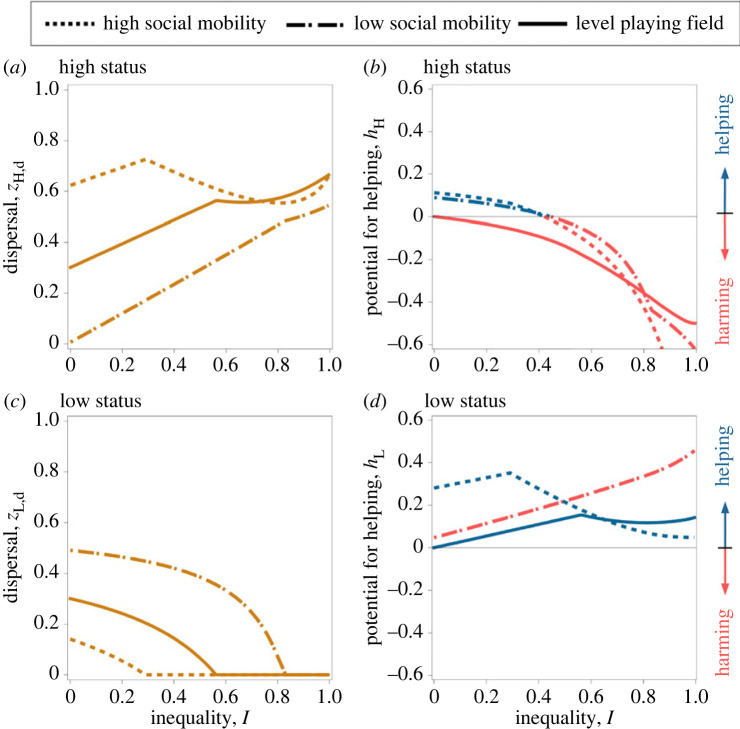
The social class with greater dispersal tends to receive more help. Optimal dispersal rates of the offspring of high- and low-status individuals (i.e. *z*_H,d*_ and *z*_L,d*_, (*a*) and (*c*), respectively), and the potential for helping by high- and low-status individuals (i.e. *h*_H_ and *h*_L_, (*b*) and (*d*), respectively) as a function of inequality (*I*) for a level playing field (i.e. *M* = 0; solid lines), maternal rank inheritance (i.e. *M* = 0.25; dashed lines) and rank-reversal inheritance (i.e. *M* = 0.75; dotted lines). Parameter values: *k* = 0.5, *n*_H_ = 1, *n*_L_ = 2.

**Figure 5.**
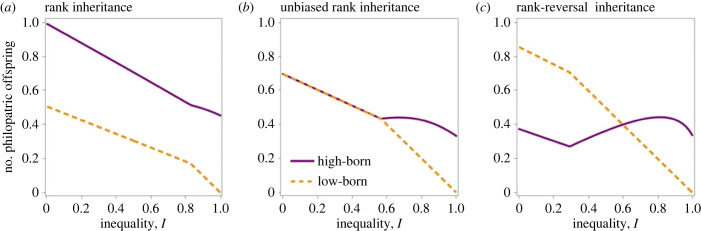
The mechanism of quality inheritance modulates status-dependent offspring philopatry. Under unbiased inheritance (level playing field, *M* = 0.5), both high- and low-status mothers produce an equal number of philopatric offspring, referred to as the Constant Philopater Hypothesis [48]. By contrast, rank inheritance (low social mobility, *M* = 0.25) results in high-status philopatry, while rank-reversal inheritance (high social mobility, *M* = 0.75) tends to result in low-status philopatry. Parameter values: *k* = 0.5, *n*_H_ = 1, *n*_L_ = 2.

When offspring adjust their dispersal behaviour according to their parent's status, we find that high-born offspring are more inclined to disperse than low-born offspring when status transmission is unbiased. This disparity arises because high-status families face greater kin competition than their low-status counterparts (figure 4*a,c*). Moreover, despite the disparity in dispersal rates between the two classes, both families ultimately produce exactly the same number of philopatric offspring irrespective of their fertility (figure 5*b*). Thus, we recover the Constant Philopater Hypothesis as proposed by Rodrigues & Gardner [48]. While this phenomenon tends to equalize the intensity of kin competition between the two families, each low-born offspring has a greater tendency to remain in the local patch compared with high-born offspring, and therefore the former end up contributing more to the competition for local resources. Consequently, high-status individuals are favoured to invest heavily in harming behaviour, such as reproductive suppression through aggression, to alleviate the competition that their own offspring face from the less mobile, low-status offspring (figure 4*b*).

#### Main prediction 3

When rank-reversal inheritance is relatively high (*M* > 1/2), low-born offspring enjoy a home-ground advantage. Thus, while these offspring have an incentive to stay in the native patch, high-born offspring are incentivized to disperse away from the patch (figure 4*a,c*). Strong rank-reversal inheritance can result in a relatively peaceful and cooperative population.

When inequality is relatively low (lower *I*), both high- and low-status individuals are favoured to invest in helping behaviour (figure 4*b,d*). Low-status individuals help high-status individuals because this leads to little kin competition, as the additional high-born offspring created in virtue of her behaviour are unlikely to remain philopatric and compete for local resources. High-status individuals help low-status individuals because this incurs relatively minor kin competition costs. Suppressing the reproductive success of low-status individuals is of little utility to high-status individuals, as their own offspring are unlikely to compete for local resources. However, when inequality rises, the disproportional production of high-born offspring contributes a significant fraction of kin competition despite their relatively higher dispersal rates. As a result of this increase in the level of competition experienced by high-status offspring, high-status individuals have an incentive to suppress the reproduction of low-status individuals through harming.

#### Main prediction 4

Under strong transmission of status, high-born offspring enjoy a competitive advantage, and therefore they show relatively low dispersal rates, in contrast with the low-born offspring who show relatively high dispersal rates (figure 4*a,c*). Status-dependent dispersal and lower fertility inequality promote within-group cooperation (figure 4*b,d*).

Under low fertility inequality (lower *I*), because low-status families are highly dispersive, they contribute little to local competition, and therefore high-status individuals are selected to help low-status individuals (*h*_H_ > 0; figure 4*b*). In addition, because low-status families experience little kin competition, low-status individuals are also favoured to invest in helping (*h*_L_ > 0; figure 4*d*). When fertility inequality rises (higher *I*), low-status individuals produce more philopatric offspring, which increases local competition. As a result, high-status individuals increasingly invest in harming behaviour to suppress the competition faced by their own offspring from those of low-status families.

We find that both rank inheritance and rank-reversal inheritance disrupt the Constant Philopater Hypothesis that arises in populations with unbiased rank inheritance [48]. Specifically, while rank inheritance promotes high-status philopatry, rank-reversal inheritance tends to favour low-status philopatry (figure 5).

## 4. Discussion

Population viscosity is a crucial mechanism for the evolution of altruism [1,2,5–8]. Recent work has sought to understand the impact of population viscosity on the evolution of social behaviour when populations are heterogeneous with respect to individual quality [10–14]. However, while these studies have not explored inheritance of quality from parent to offspring, such effects are widespread in the natural world and can be observed in multiple taxa, from insects to mammals, including primates and humans (e.g. [19–21,29,36,44,45,60–62]). Here, we have studied how correlations between parental and offspring quality, in the form of social status, influence the impact of population viscosity on the evolution of helping and harming.

We have found that the degree of status inheritance (and its complement, the degree of social mobility) greatly impacts the evolution of helping and harming. When there is high transmission of social status (low social mobility) from parents to offspring, natural selection favours low-status individuals to invest more heavily into helping high-status individuals, and high-status individuals to refrain from harming low-status individuals. This means that linear transmission of status promotes relatively more cooperative groups, with more cooperation being associated with an increase in group productivity. Status-reversal transmission has a more complex effect on social roles. When societies are characterized by low inequality in fertility but status-reversal transmission, social behaviour of high-status individuals is reversed. That is, high-status individuals now invest a considerable share of their resources into helping behaviour, rather than harming behaviour. By contrast, when societies are characterized by high inequality in fertility and status-reversal transmission, high-status individuals are selected to invest heavily into harming behaviour, and low-status individuals are selected to reduce their investment into helping behaviour. Thus, we find that high inequality in fertility in conjunction with status-reversal transmission is conducive to within-group conflict and an associated decrease in group productivity.

In more complex organisms, juveniles may be able to adjust their dispersal behaviour according to the status of their parents, in which cases, the patterns of investment into helping and harming behaviour change considerably. Because high- and low-born juveniles show distinct patterns of dispersal, the intensity of kin competition experienced varies with the status of the family. Maternal transmission of status, for instance, discourages high-born offspring from leaving their native patch, but encourages low-born offspring to leave their native patch. Philopatry of high-born juveniles and migration of low-born juveniles increase the genetic structuring in the population, which causes high relatedness within groups. Because low-born offspring leave their native patch, helping behaviours by high-status breeders that create additional low-born offspring do not carry a corresponding increment in local kin competition costs. The co-occurrence of these two phenomena—high relatedness on the one hand, and low cooperation-induced kin competition, on the other hand—under maternal rank inheritance promotes cooperative behaviour by high-status breeders.

Our model predicts that mechanisms of status transmission lead to offspring philopatry when dispersal involves the loss of social support and its associated competitive advantages. This pattern aligns with observed phenomena in multiple species. For instance, in southern pied babblers (*Turdoides bicolor*), low-status males are more likely to disperse than their high-status counterparts [63]. In spotted hyaenas (*Crocuta crocuta*), female dominance emerges from maternal social support, which in turn arises from female philopatry [50]. Additionally, in pre-industrial Finland, male heirs among landowning families had a higher than average likelihood of remaining philopatric, whereas this effect was absent in landless families [64]. Furthermore, our study shows that mechanisms of status transmission can counteract the kin competition forces that underlie the Constant Philopater Hypothesis [48], disrupting the unbiased-philopatry invariant. Thus, measuring the intensity of kin competition alone may be insufficient to predict patterns of philopatry (e.g. [65]). Our findings also highlight that status-dependent philopatry can emerge through a wide range of mechanisms, including maternal support, even without direct status-dependent control over dispersal phenotypes [48]. These results emphasize the intricate nature of selective pressures and their contrasting effects on dispersal evolution within social groups, as illustrated by the empirical work of Nitsch showing that understanding dispersal phenotypes requires careful accounting of multiple factors, including the land-owning status of families, mechanisms of transmission of status and the intensity of kin competition [64,65].

Overall, we find that once mechanisms of status transmission are in place, stratification becomes more stable. Rank across generations becomes less stable when higher-status individuals are less willing to defend their status, while lower-status individuals are more willing to fight for status. The range of parameter values under which this combination of behaviours evolved is extremely narrow. The evolution of negative or competitive status-seeking behaviours (i.e. harming by subordinate individuals) requires: (1) status to be associated with access to valuable resources, (2) effective status-seeking behaviours and (3) individuals that directly benefit from status-seeking behaviours. The range of parameter values under which all these conditions co-occur is limited. For instance, a gradient in fertility does increase the value of high-status positions (condition 1), but it reduces the number of beneficiaries of status-seeking behaviours (condition 3). Similarly, inter-generational transmission of status does increase the value of high-status positions (condition 1), but it reduces the effectiveness of status-seeking behaviour (condition 2). Because the conditions for the evolution of status-seeking behaviours can trade-off with each other, lower levels of inequality can actually increase selection for status-seeking behaviours. This is because while lower levels of inequality decrease the value of high-status positions (condition 1), it can also increase the effectiveness of status-seeking behaviour (condition 2) and/or the number of beneficiaries of the status-seeking behaviour (condition 3).

These conditions for the evolution of competitive status-seeking (or status-keeping) behaviours can explain several empirical findings. For instance, in the wasp (*Parischnogaster mellyi*), some studies suggest that aggression is higher when fertility inequality is lower [66]. This may occur because lower inequality in fertility increases the number of offspring benefiting from maternal status-seeking behaviours due to higher levels of fertility among low-status individuals (condition 3). In meerkats, aggression by dominant individuals (competitive status-keeping behaviours) is associated with subordinate reproduction (condition 3), and subordinate reproduction is associated with lower survival of a dominant's offspring (condition 2) [67].

Many animal societies are organized in hierarchical systems, in which social status is tightly associated with contrasting suites of behavioural and life-history traits. For instance, in hyaenas, high-status individuals have preferential access to food items, are more aggressive towards their social partners, are healthier, and are more likely to produce viable young than their low-status rivals [36,68]. Several of these status-specific traits are transmitted from parents to offspring, with offspring of high-status individuals, for instance, having priority of access to high-quality food items like their parents [36,68]. Consequently, high-born offspring often grow faster, become heavier and healthier and are more likely to become high-status adults than low-born offspring [36]. In other species, like in the banded mongooses, the correlation between maternal and offspring status is weaker, if not absent [69]. Our study suggests that in societies where offspring inherit the status of their parents, relatedness among group members is higher, aggression among group members tends to be lower, and groups become more productive.

Our work holds potential implications for various areas within evolutionary biology. Previous research has shown that reproductive skew alters local relatedness among pairs of individuals [59,70]. Further studies on heterogeneous groups have revealed that the costs and benefits of cooperation depend on the reproductive value of actors and recipients [12]. More recently, research has demonstrated that heterogeneity in reproductive value affects the evolution of fairness, which is more likely to evolve when fairness is expressed by low-reproductive value individuals, and the beneficiaries are high-reproductive value relatives [71]. Our study indicates that maternal effects can mediate relatedness and the costs and benefits of cooperation, suggesting that deeper integration between maternal effects and both reproductive skew theory [72] and the evolution of fairness can yield novel insights. Furthermore, our work shows that the value of offspring depends on factors such as their dispersal status, maternal support and maternal social status. In natural populations, these factors often exhibit sex-specific patterns, which might impact the evolution of the sex ratio in ways akin to the Trivers-Willard effect [73]. Integrating these biological factors into sex ratio theory presents a promising direction for future research.

We have shown that philopatry can confer a life-history advantage over dispersal through a ‘home-ground advantage’ effect that is common in many species [15,51,62]. However, the influence of extrinsic factors on class correlations should not be overlooked [11,14], and their role in modulating cooperation warrants further investigation. In addition, some organisms acquire phenotypes early in life that confer fitness advantages regardless of their subsequent dispersal status. To explore this possibility, we have conducted a preliminary analysis of the evolution of cooperation in the appendix, which suggests that our main conclusions hold qualitatively when early-life effects persist after dispersal. However, a more comprehensive investigation of the potential interactions between different forms of inheritance and dispersal strategies would contribute to a deeper understanding of the evolutionary dynamics underlying animal societies. Our study highlights the importance of considering the different mechanisms of phenotypic inheritance and their potential interactions in shaping cooperation in animal societies.

## Data Availability

Supplementary material is available online [74].
